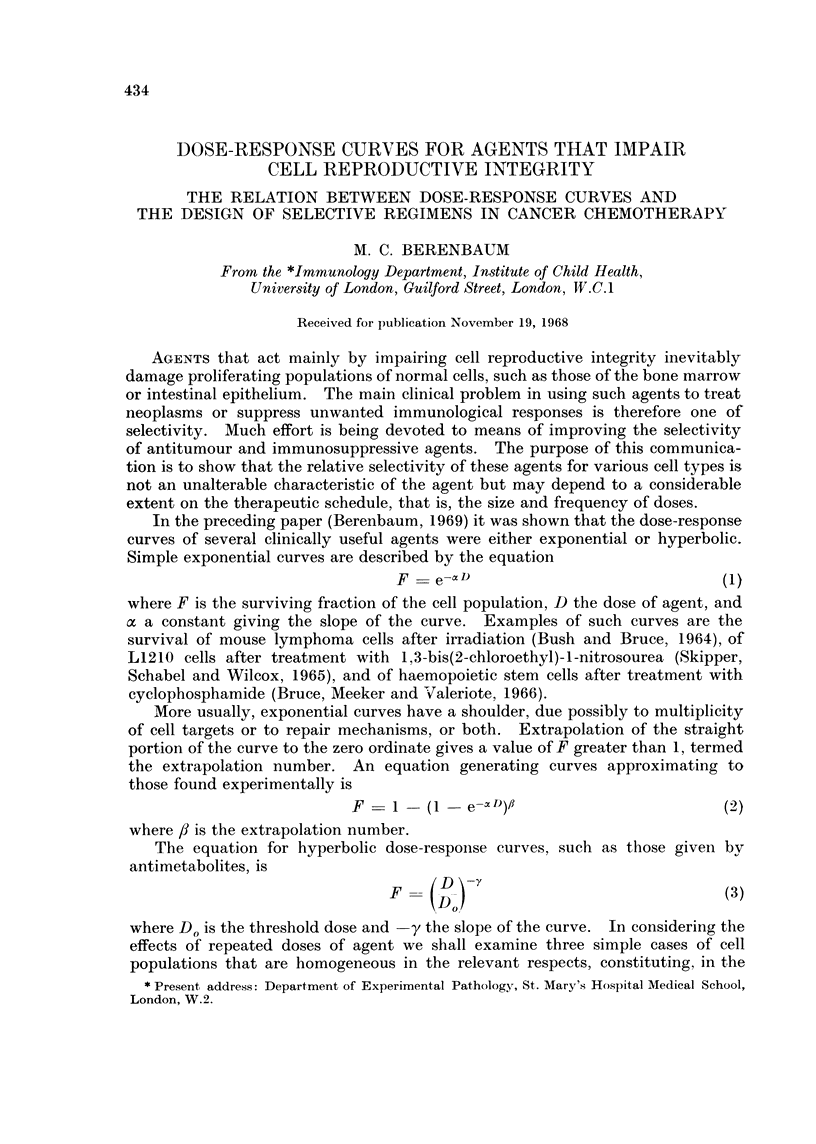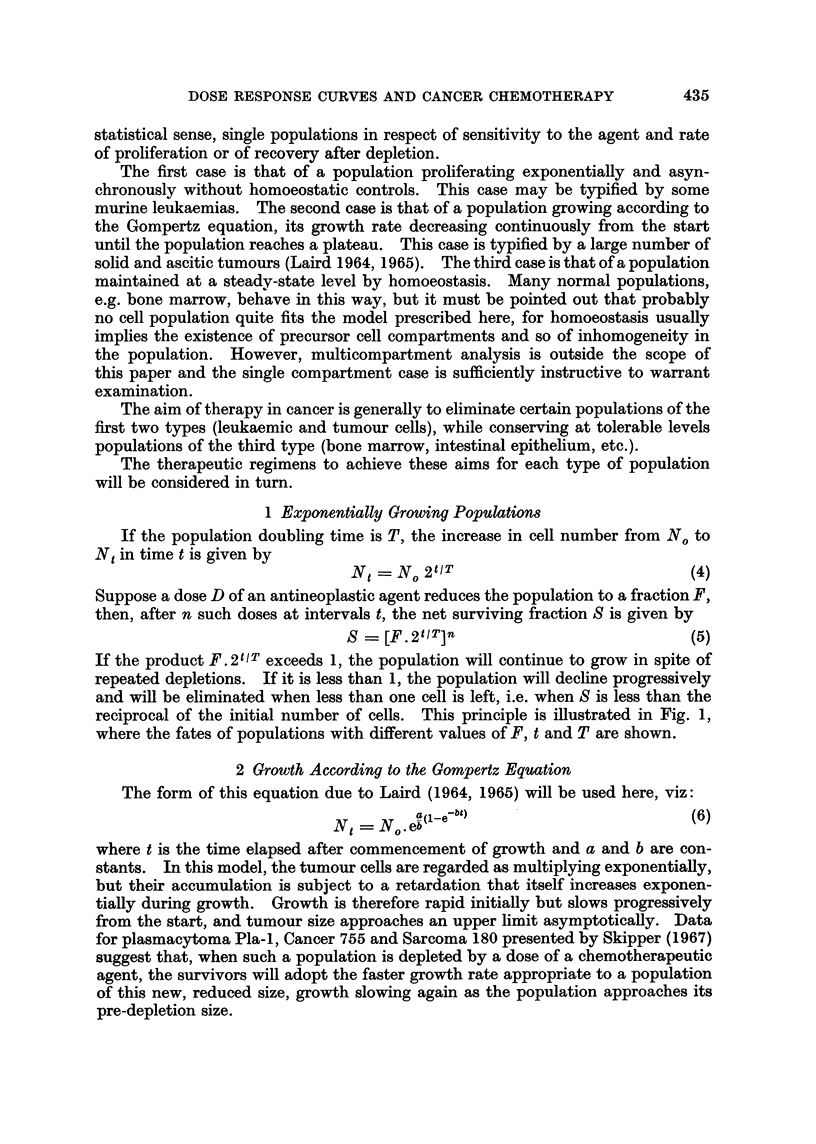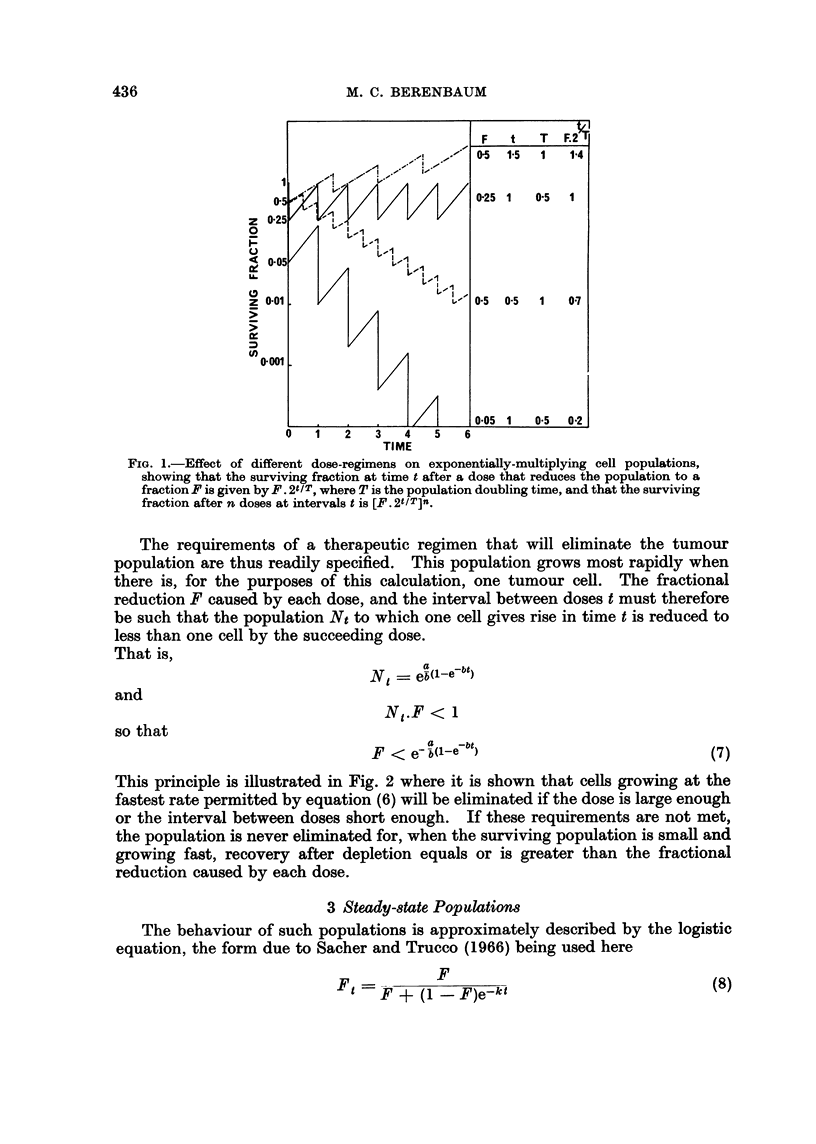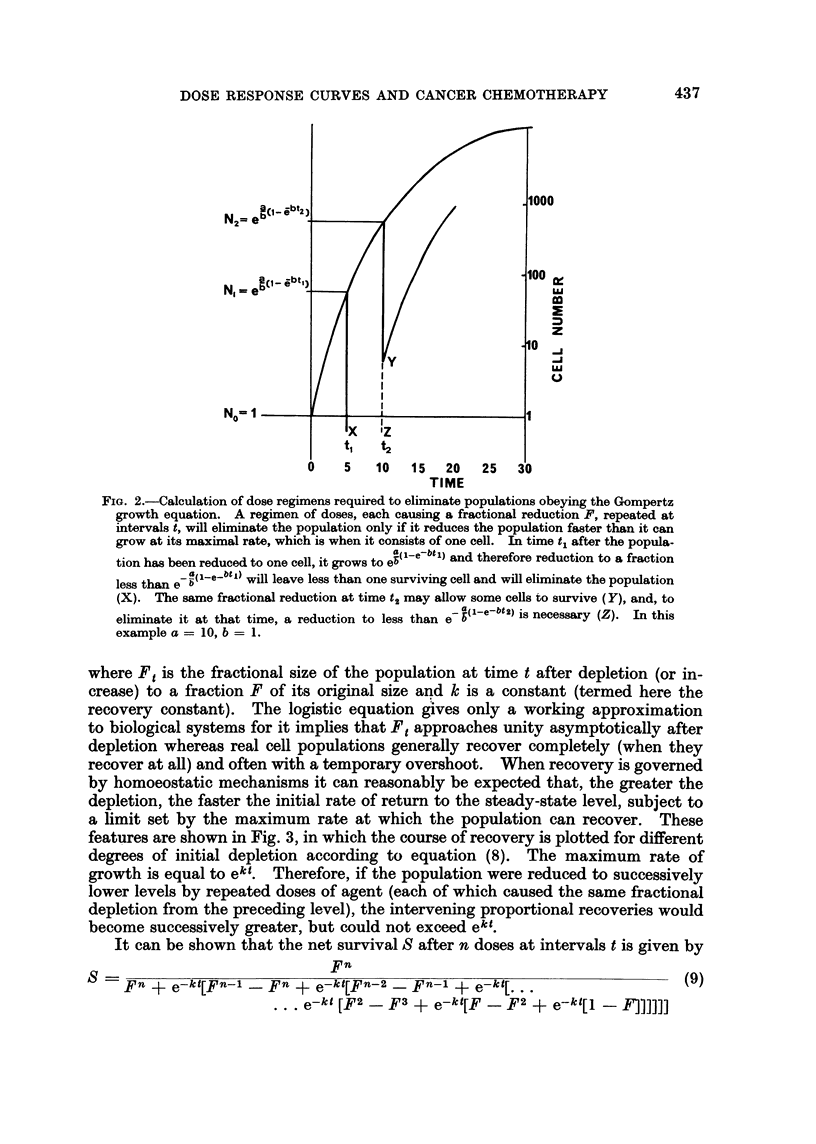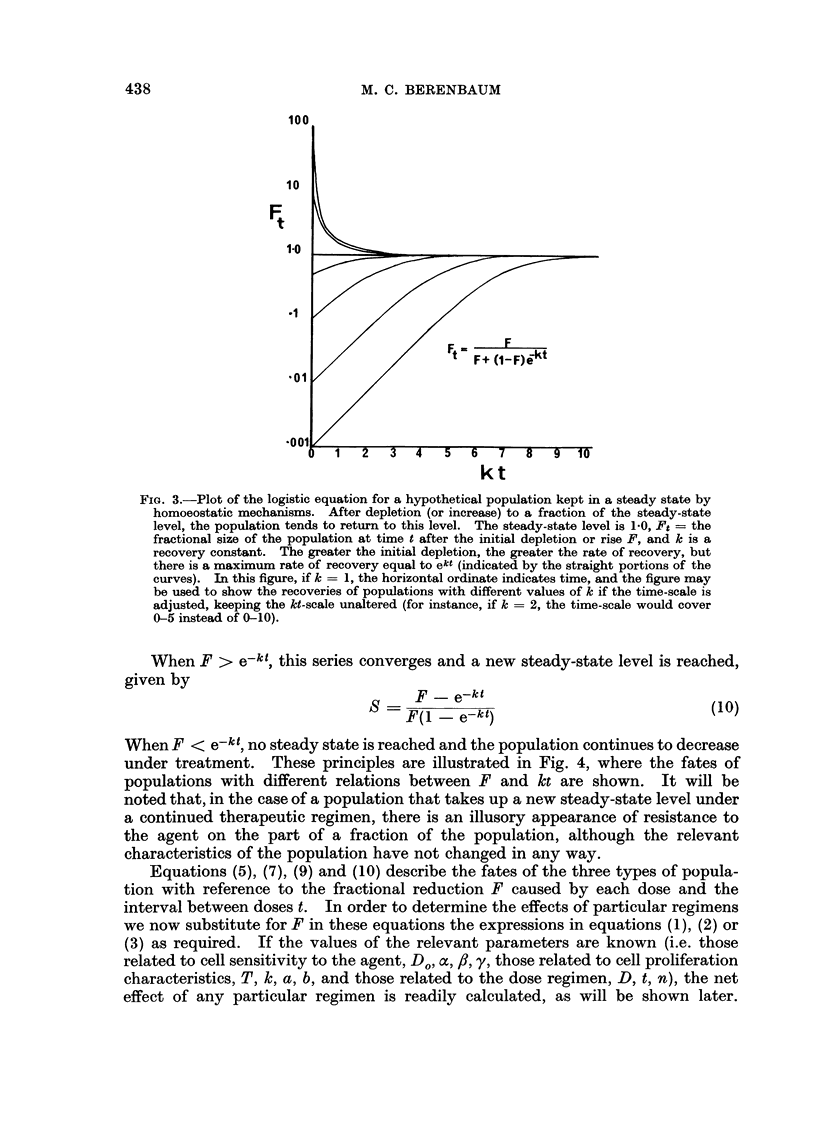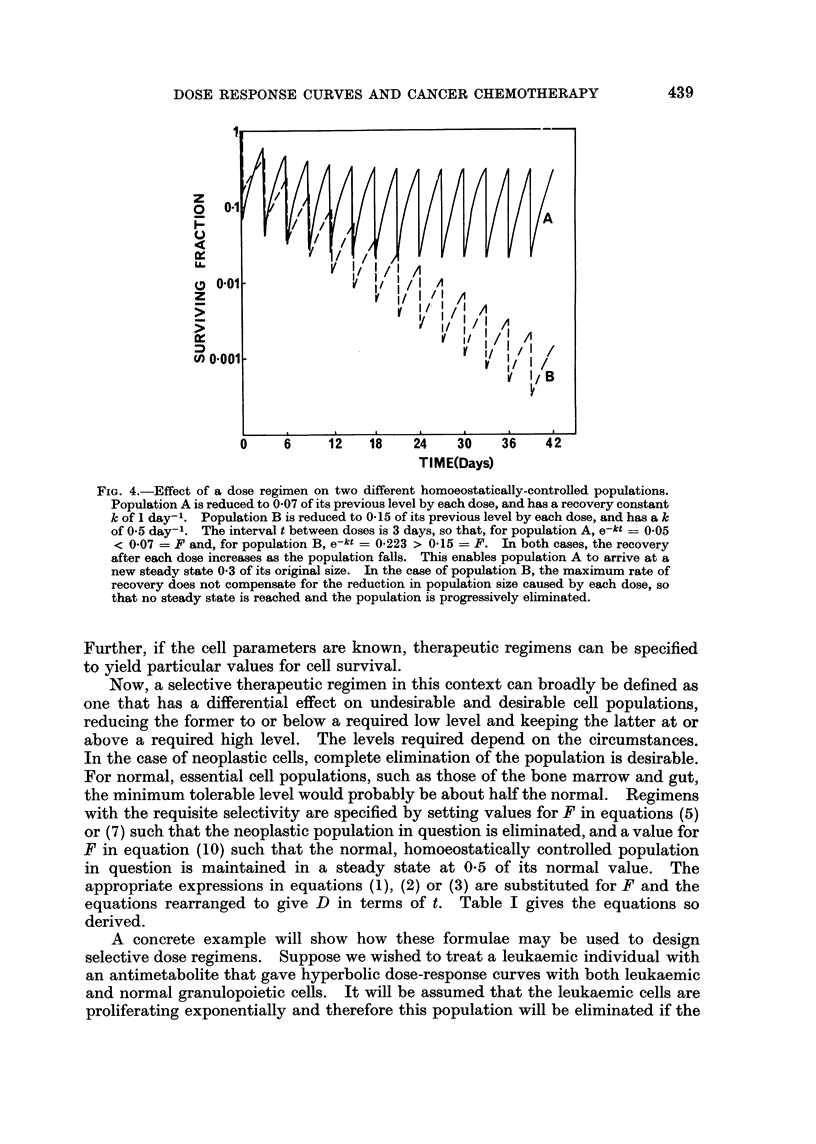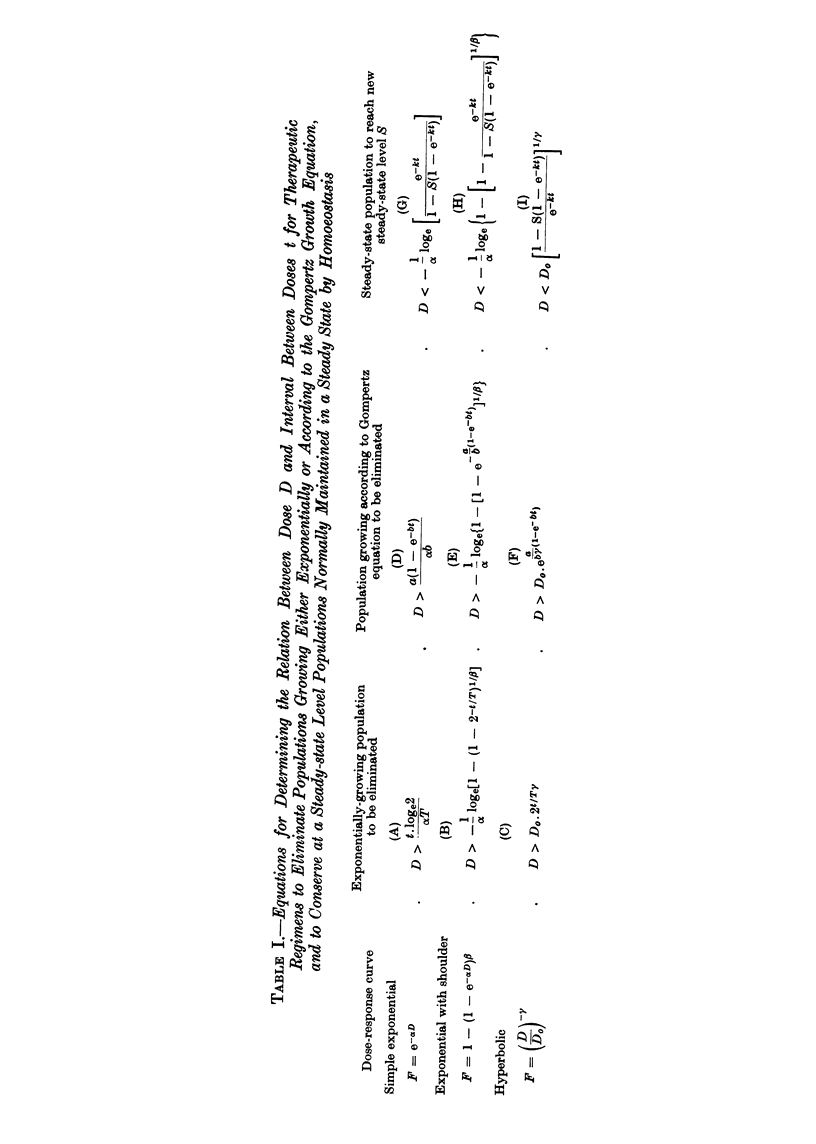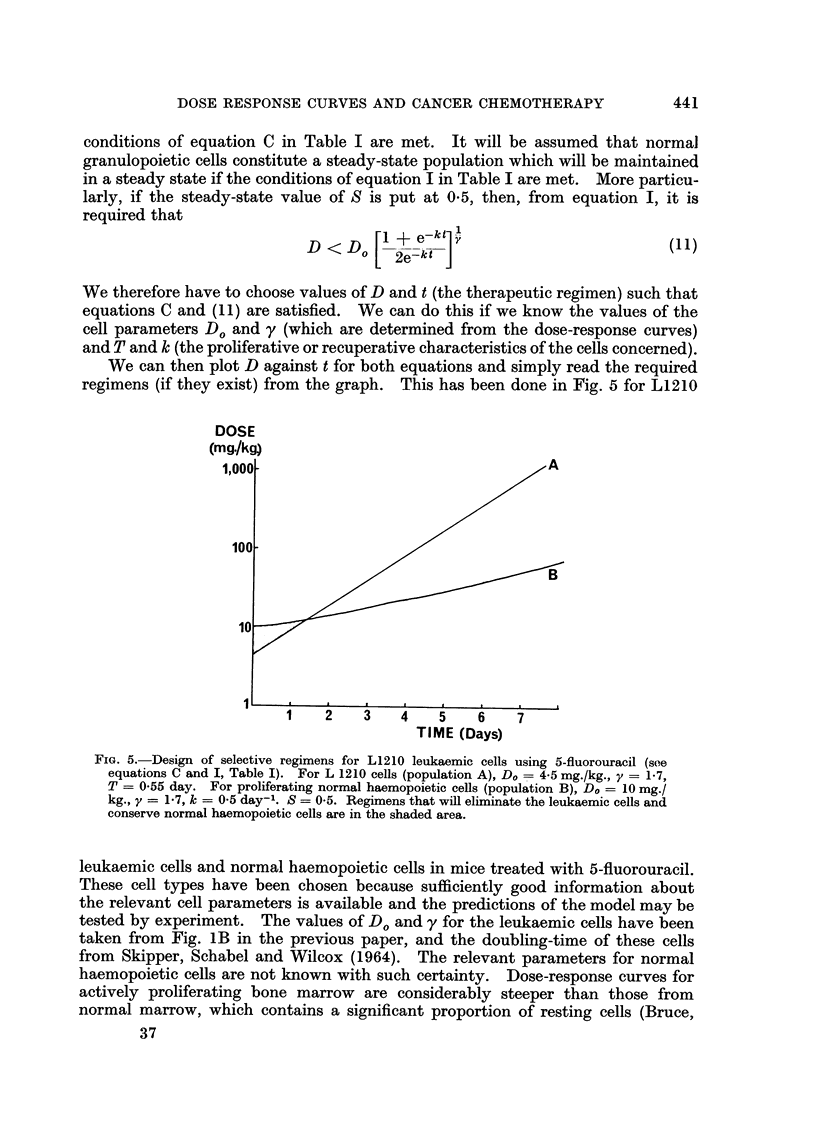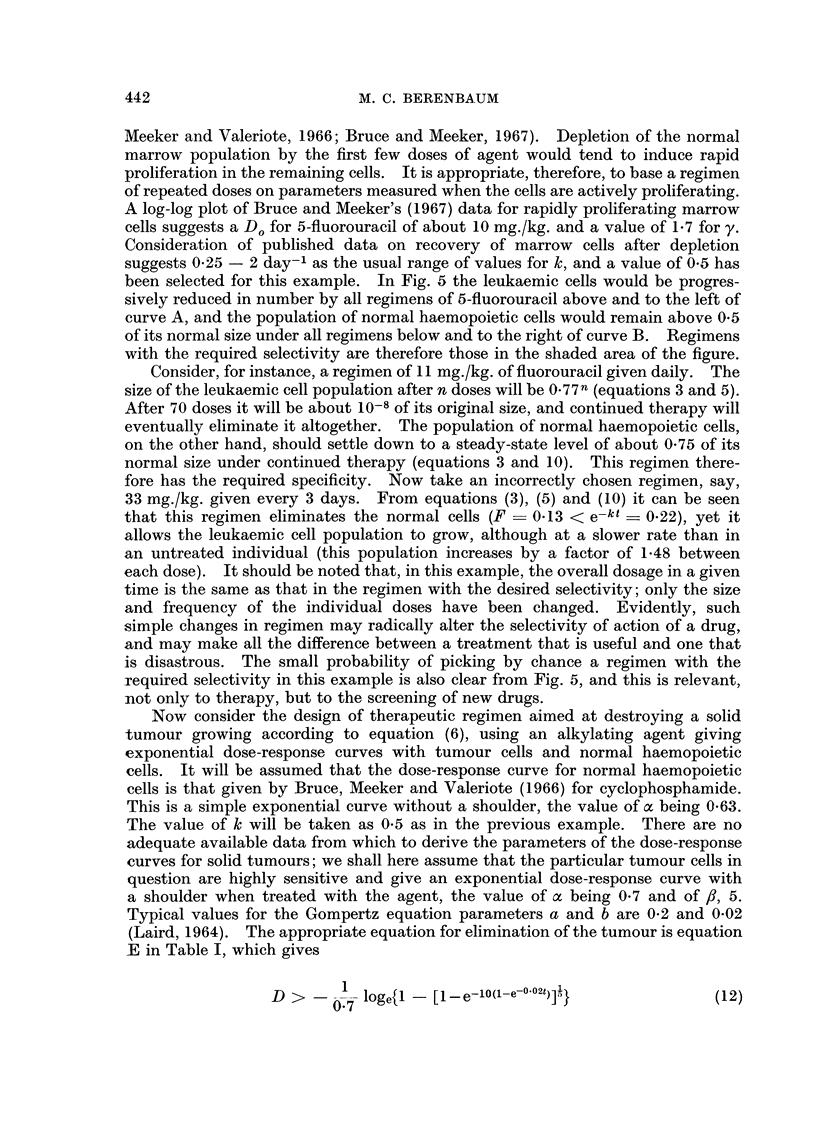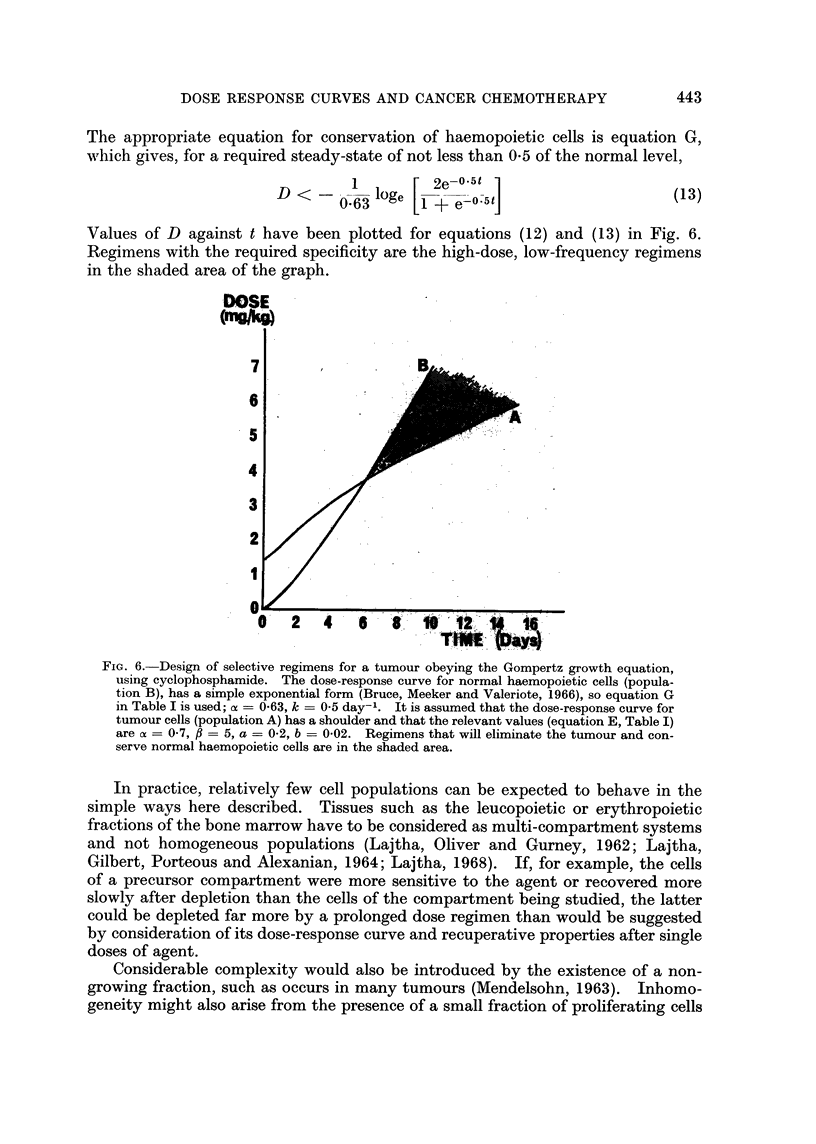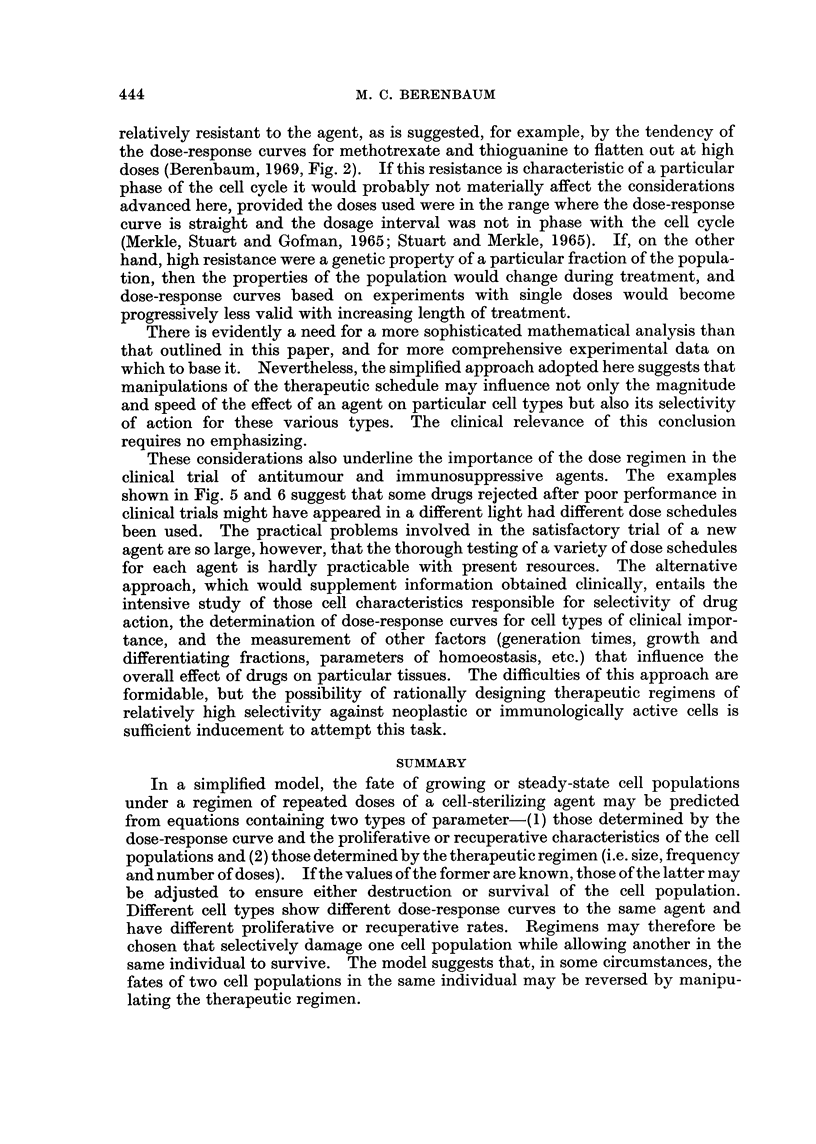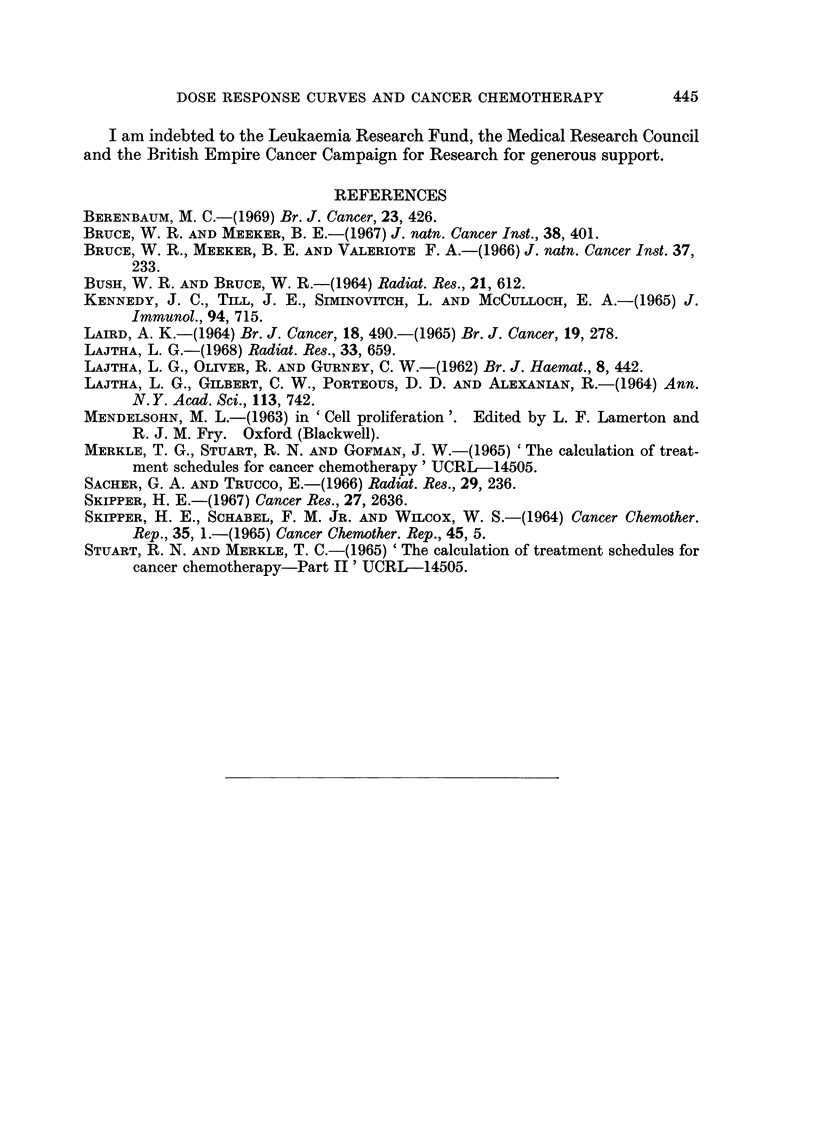# Dose-response curves for agents that impair cell reproductive integrity. The relation between dose-response curves and the design of selective regimens in cancer chemotherapy.

**DOI:** 10.1038/bjc.1969.56

**Published:** 1969-06

**Authors:** M. C. Berenbaum


### DOSE-RESPONSE CURVES FOR AGENTS THAT IMPAIR CELL REPRODUCTIVE INTEGRITY

### THE RELATION BETWEEN DOSE-RESPONSE CURVES AND THE DESIGN OF SELECTIVE REGIMENS IN CANCER CHEMOTHERAPY

M. C. BERENBAUM

*From the *Immunology Department, Institute of Child Health, University of London, Guilford Street, London, W.C.*1

Received for publication November 19, 1968

AGENTS that act mainly by impairing cell reproductive integrity inevitably damage proliferating populations of normal cells, such as those of the bone marrow or intestinal epithelium. The main clinical problem in using such agents to treat neoplasms or suppress unwanted immunological responses is therefore one of selectivity. Much effort is being devoted to means of improving the selectivity of antitumour and immunosuppressive agents. The purpose of this communication is to show that the relative selectivity of these agents for various cell types is not an unalterable characteristic of the agent but may depend to a considerable extent on the therapeutic schedule, that is, the size and frequency of doses.

In the preceding paper (Berenbaum, 1969) it was shown that the dose-response curves of several clinically useful agents were either exponential or hyperbolic. Simple exponential curves are described by the equation

$$F = e^{-\alpha D} \tag{1}$$

where $F$ is the surviving fraction of the cell population, $D$ the dose of agent, and $\alpha$ a constant giving the slope of the curve. Examples of such curves are the survival of mouse lymphoma cells after irradiation (Bush and Bruce, 1964), of L1210 cells after treatment with 1,3-bis(2-chloroethyl)-1-nitrosourea (Skipper, Schabel and Wilcox, 1965), and of haemopoietic stem cells after treatment with cyclophosphamide (Bruce, Meeker and Valeriote, 1966).

More usually, exponential curves have a shoulder, due possibly to multiplicity of cell targets or to repair mechanisms, or both. Extrapolation of the straight portion of the curve to the zero ordinate gives a value of $F$ greater than 1, termed the extrapolation number. An equation generating curves approximating to those found experimentally is

$$F = 1 - (1 - e^{-\alpha D})^{\beta} \tag{2}$$

where $\beta$ is the extrapolation number.

The equation for hyperbolic dose-response curves, such as those given by antimetabolites, is

$$F = \left(\frac{D}{D_o}\right)^{-\gamma} \tag{3}$$

where $D_0$ is the threshold dose and $-\gamma$ the slope of the curve. In considering the effects of repeated doses of agent we shall examine three simple cases of cell populations that are homogeneous in the relevant respects, constituting, in the

* Present address: Department of Experimental Pathology, St. Mary's Hospital Medical School, London, W.2.

statistical sense, single populations in respect of sensitivity to the agent and rate of proliferation or of recovery after depletion.

The first case is that of a population proliferating exponentially and asynchronously without homoeostatic controls. This case may be typified by some murine leukaemias. The second case is that of a population growing according to the Gompertz equation, its growth rate decreasing continuously from the start until the population reaches a plateau. This case is typified by a large number of solid and ascitic tumours (Laird 1964, 1965). The third case is that of a population maintained at a steady-state level by homoeostasis. Many normal populations, e.g. bone marrow, behave in this way, but it must be pointed out that probably no cell population quite fits the model prescribed here, for homoeostasis usually implies the existence of precursor cell compartments and so of inhomogeneity in the population. However, multicompartment analysis is outside the scope of this paper and the single compartment case is sufficiently instructive to warrant examination.

The aim of therapy in cancer is generally to eliminate certain populations of the first two types (leukaemic and tumour cells), while conserving at tolerable levels populations of the third type (bone marrow, intestinal epithelium, etc.).

The therapeutic regimens to achieve these aims for each type of population will be considered in turn.

### 1 *Exponentially Growing Populations*

If the population doubling time is $T$, the increase in cell number from $N_o$ to $N_t$ in time $t$ is given by

$$N_t = N_o\, 2^{t/T} \qquad (4)$$

Suppose a dose $D$ of an antineoplastic agent reduces the population to a fraction $F$, then, after $n$ such doses at intervals $t$, the net surviving fraction $S$ is given by

$$S = [F\,.\,2^{t/T}]^n \qquad (5)$$

If the product $F\,.\,2^{t/T}$ exceeds 1, the population will continue to grow in spite of repeated depletions. If it is less than 1, the population will decline progressively and will be eliminated when less than one cell is left, i.e. when $S$ is less than the reciprocal of the initial number of cells. This principle is illustrated in Fig. 1, where the fates of populations with different values of $F$, $t$ and $T$ are shown.

### 2 *Growth According to the Gompertz Equation*

The form of this equation due to Laird (1964, 1965) will be used here, viz:

$$N_t = N_o\,.\,e^{\frac{a}{b}(1-e^{-bt})} \qquad (6)$$

where $t$ is the time elapsed after commencement of growth and $a$ and $b$ are constants. In this model, the tumour cells are regarded as multiplying exponentially, but their accumulation is subject to a retardation that itself increases exponentially during growth. Growth is therefore rapid initially but slows progressively from the start, and tumour size approaches an upper limit asymptotically. Data for plasmacytoma Pla-1, Cancer 755 and Sarcoma 180 presented by Skipper (1967) suggest that, when such a population is depleted by a dose of a chemotherapeutic agent, the survivors will adopt the faster growth rate appropriate to a population of this new, reduced size, growth slowing again as the population approaches its pre-depletion size.

FIG. 1.—Effect of different dose-regimens on exponentially-multiplying cell populations, showing that the surviving fraction at time $t$ after a dose that reduces the population to a fraction $F$ is given by $F.2^{t/T}$, where $T$ is the population doubling time, and that the surviving fraction after $n$ doses at intervals $t$ is $[F.2^{t/T}]^n$.

The requirements of a therapeutic regimen that will eliminate the tumour population are thus readily specified. This population grows most rapidly when there is, for the purposes of this calculation, one tumour cell. The fractional reduction $F$ caused by each dose, and the interval between doses $t$ must therefore be such that the population $N_t$ to which one cell gives rise in time $t$ is reduced to less than one cell by the succeeding dose.
That is,

$$N_t = e^{\frac{a}{b}(1-e^{-bt})}$$

and

$$N_t.F < 1$$

so that

$$F < e^{-\frac{a}{b}(1-e^{-bt})} \tag{7}$$

This principle is illustrated in Fig. 2 where it is shown that cells growing at the fastest rate permitted by equation (6) will be eliminated if the dose is large enough or the interval between doses short enough. If these requirements are not met, the population is never eliminated for, when the surviving population is small and growing fast, recovery after depletion equals or is greater than the fractional reduction caused by each dose.

### 3 *Steady-state Populations*

The behaviour of such populations is approximately described by the logistic equation, the form due to Sacher and Trucco (1966) being used here

$$F_t = \frac{F}{F + (1 - F)e^{-kt}} \tag{8}$$

FIG. 2.—Calculation of dose regimens required to eliminate populations obeying the Gompertz growth equation. A regimen of doses, each causing a fractional reduction $F$, repeated at intervals $t$, will eliminate the population only if it reduces the population faster than it can grow at its maximal rate, which is when it consists of one cell. In time $t_1$ after the population has been reduced to one cell, it grows to $e^{\frac{a}{b}(1-e^{-bt_1})}$ and therefore reduction to a fraction less than $e^{-\frac{a}{b}(1-e^{-bt_1})}$ will leave less than one surviving cell and will eliminate the population (X). The same fractional reduction at time $t_2$ may allow some cells to survive ($Y$), and, to eliminate it at that time, a reduction to less than $e^{-\frac{a}{b}(1-e^{-bt_2})}$ is necessary ($Z$). In this example $a = 10$, $b = 1$.

where $F_t$ is the fractional size of the population at time $t$ after depletion (or increase) to a fraction $F$ of its original size and $k$ is a constant (termed here the recovery constant). The logistic equation gives only a working approximation to biological systems for it implies that $F_t$ approaches unity asymptotically after depletion whereas real cell populations generally recover completely (when they recover at all) and often with a temporary overshoot. When recovery is governed by homoeostatic mechanisms it can reasonably be expected that, the greater the depletion, the faster the initial rate of return to the steady-state level, subject to a limit set by the maximum rate at which the population can recover. These features are shown in Fig. 3, in which the course of recovery is plotted for different degrees of initial depletion according to equation (8). The maximum rate of growth is equal to $e^{kt}$. Therefore, if the population were reduced to successively lower levels by repeated doses of agent (each of which caused the same fractional depletion from the preceding level), the intervening proportional recoveries would become successively greater, but could not exceed $e^{kt}$.

It can be shown that the net survival $S$ after $n$ doses at intervals $t$ is given by

$$S = \frac{F^n}{F^n + e^{-kt}[F^{n-1} - F^n + e^{-kt}[F^{n-2} - F^{n-1} + e^{-kt}[\ldots \ldots e^{-kt}[F^2 - F^3 + e^{-kt}[F - F^2 + e^{-kt}[1 - F]]]]]]} \qquad (9)$$

FIG. 3.—Plot of the logistic equation for a hypothetical population kept in a steady state by homoeostatic mechanisms. After depletion (or increase) to a fraction of the steady-state level, the population tends to return to this level. The steady-state level is 1·0, $F_t$ = the fractional size of the population at time $t$ after the initial depletion or rise $F$, and $k$ is a recovery constant. The greater the initial depletion, the greater the rate of recovery, but there is a maximum rate of recovery equal to $e^{kt}$ (indicated by the straight portions of the curves). In this figure, if $k = 1$, the horizontal ordinate indicates time, and the figure may be used to show the recoveries of populations with different values of $k$ if the time-scale is adjusted, keeping the $kt$-scale unaltered (for instance, if $k = 2$, the time-scale would cover 0–5 instead of 0–10).

When $F > e^{-kt}$, this series converges and a new steady-state level is reached, given by

$$S = \frac{F - e^{-kt}}{F(1 - e^{-kt})} \tag{10}$$

When $F < e^{-kt}$, no steady state is reached and the population continues to decrease under treatment. These principles are illustrated in Fig. 4, where the fates of populations with different relations between $F$ and $kt$ are shown. It will be noted that, in the case of a population that takes up a new steady-state level under a continued therapeutic regimen, there is an illusory appearance of resistance to the agent on the part of a fraction of the population, although the relevant characteristics of the population have not changed in any way.

Equations (5), (7), (9) and (10) describe the fates of the three types of population with reference to the fractional reduction $F$ caused by each dose and the interval between doses $t$. In order to determine the effects of particular regimens we now substitute for $F$ in these equations the expressions in equations (1), (2) or (3) as required. If the values of the relevant parameters are known (i.e. those related to cell sensitivity to the agent, $D_0$, $\alpha$, $\beta$, $\gamma$, those related to cell proliferation characteristics, $T$, $k$, $a$, $b$, and those related to the dose regimen, $D$, $t$, $n$), the net effect of any particular regimen is readily calculated, as will be shown later.

FIG. 4.—Effect of a dose regimen on two different homoeostatically-controlled populations. Population A is reduced to 0·07 of its previous level by each dose, and has a recovery constant $k$ of 1 day$^{-1}$. Population B is reduced to 0·15 of its previous level by each dose, and has a $k$ of 0·5 day$^{-1}$. The interval $t$ between doses is 3 days, so that, for population A, $e^{-kt} = 0{\cdot}05 < 0{\cdot}07 = F$ and, for population B, $e^{-kt} = 0{\cdot}223 > 0{\cdot}15 = F$. In both cases, the recovery after each dose increases as the population falls. This enables population A to arrive at a new steady state 0·3 of its original size. In the case of population B, the maximum rate of recovery does not compensate for the reduction in population size caused by each dose, so that no steady state is reached and the population is progressively eliminated.

Further, if the cell parameters are known, therapeutic regimens can be specified to yield particular values for cell survival.

Now, a selective therapeutic regimen in this context can broadly be defined as one that has a differential effect on undesirable and desirable cell populations, reducing the former to or below a required low level and keeping the latter at or above a required high level. The levels required depend on the circumstances. In the case of neoplastic cells, complete elimination of the population is desirable. For normal, essential cell populations, such as those of the bone marrow and gut, the minimum tolerable level would probably be about half the normal. Regimens with the requisite selectivity are specified by setting values for $F$ in equations (5) or (7) such that the neoplastic population in question is eliminated, and a value for $F$ in equation (10) such that the normal, homoeostatically controlled population in question is maintained in a steady state at 0·5 of its normal value. The appropriate expressions in equations (1), (2) or (3) are substituted for $F$ and the equations rearranged to give $D$ in terms of $t$. Table I gives the equations so derived.

A concrete example will show how these formulae may be used to design selective dose regimens. Suppose we wished to treat a leukaemic individual with an antimetabolite that gave hyperbolic dose-response curves with both leukaemic and normal granulopoietic cells. It will be assumed that the leukaemic cells are proliferating exponentially and therefore this population will be eliminated if the

TABLE I.—*Equations for Determining the Relation Between Dose D and Interval Between Doses t for Therapeutic Regimens to Eliminate Populations Growing Either Exponentially or According to the Gompertz Growth Equation, and to Conserve at a Steady-state Level Populations Normally Maintained in a Steady State by Homoeostasis*

| Dose-response curve | Exponentially-growing population to be eliminated | Population growing according to Gompertz equation to be eliminated | Steady-state population to reach new steady-state level $S$ |
|---|---|---|---|
| Simple exponential | (A) | (D) | (G) |
| $F = e^{-\alpha D}$ . | $D > \frac{t . \log_e 2}{\alpha T}$ . | $D > \frac{a(1 - e^{-bt})}{\alpha b}$ . | $D < -\frac{1}{\alpha} \log_e \left[\frac{e^{-kt}}{1 - S(1 - e^{-kt})}\right]$ |
| Exponential with shoulder | (B) | (E) | (H) |
| $F = 1 - (1 - e^{-\alpha D})^{\beta}$ . | $D > -\frac{1}{\alpha} \log_e[1 - (1 - 2^{-t/T})^{1/\beta}]$ . | $D > -\frac{1}{\alpha} \log_e\{1 - [1 - e^{-\frac{a}{b}(1-e^{-bt})}]^{1/\beta}\}$ . | $D < -\frac{1}{\alpha} \log_e \left\{1 - \left[1 - \frac{e^{-kt}}{1 - S(1 - e^{-kt})}\right]^{1/\beta}\right\}$ |
| Hyperbolic | (C) | (F) | (I) |
| $F = \left(\frac{D}{D_o}\right)^{-\gamma}$ . | $D > D_o . 2^{t/T\gamma}$ . | $D > D_o . e^{\frac{a}{b\gamma}(1-e^{-bt})}$ . | $D < D_o \left[\frac{1 - S(1 - e^{-kt})}{e^{-kt}}\right]^{1/\gamma}$ |

conditions of equation C in Table I are met. It will be assumed that normal granulopoietic cells constitute a steady-state population which will be maintained in a steady state if the conditions of equation I in Table I are met. More particularly, if the steady-state value of $S$ is put at 0·5, then, from equation I, it is required that

$$D < D_o \left[\frac{1 + e^{-kt}}{2e^{-kt}}\right]^{\frac{1}{\gamma}} \tag{11}$$

We therefore have to choose values of $D$ and $t$ (the therapeutic regimen) such that equations C and (11) are satisfied. We can do this if we know the values of the cell parameters $D_0$ and $\gamma$ (which are determined from the dose-response curves) and $T$ and $k$ (the proliferative or recuperative characteristics of the cells concerned).

We can then plot $D$ against $t$ for both equations and simply read the required regimens (if they exist) from the graph. This has been done in Fig. 5 for L1210

FIG. 5.—Design of selective regimens for L1210 leukaemic cells using 5-fluorouracil (see equations C and I, Table I). For L 1210 cells (population A), $D_o$ = 4·5 mg./kg., $\gamma$ = 1·7, $T$ = 0·55 day. For proliferating normal haemopoietic cells (population B), $D_o$ = 10 mg./kg., $\gamma$ = 1·7, $k$ = 0·5 day$^{-1}$. $S$ = 0·5. Regimens that will eliminate the leukaemic cells and conserve normal haemopoietic cells are in the shaded area.

leukaemic cells and normal haemopoietic cells in mice treated with 5-fluorouracil. These cell types have been chosen because sufficiently good information about the relevant cell parameters is available and the predictions of the model may be tested by experiment. The values of $D_0$ and $\gamma$ for the leukaemic cells have been taken from Fig. 1B in the previous paper, and the doubling-time of these cells from Skipper, Schabel and Wilcox (1964). The relevant parameters for normal haemopoietic cells are not known with such certainty. Dose-response curves for actively proliferating bone marrow are considerably steeper than those from normal marrow, which contains a significant proportion of resting cells (Bruce,

Meeker and Valeriote, 1966; Bruce and Meeker, 1967). Depletion of the normal marrow population by the first few doses of agent would tend to induce rapid proliferation in the remaining cells. It is appropriate, therefore, to base a regimen of repeated doses on parameters measured when the cells are actively proliferating. A log-log plot of Bruce and Meeker's (1967) data for rapidly proliferating marrow cells suggests a $D_0$ for 5-fluorouracil of about 10 mg./kg. and a value of 1·7 for $\gamma$. Consideration of published data on recovery of marrow cells after depletion suggests 0·25 — 2 day$^{-1}$ as the usual range of values for $k$, and a value of 0·5 has been selected for this example. In Fig. 5 the leukaemic cells would be progressively reduced in number by all regimens of 5-fluorouracil above and to the left of curve A, and the population of normal haemopoietic cells would remain above 0·5 of its normal size under all regimens below and to the right of curve B. Regimens with the required selectivity are therefore those in the shaded area of the figure.

Consider, for instance, a regimen of 11 mg./kg. of fluorouracil given daily. The size of the leukaemic cell population after $n$ doses will be $0{\cdot}77^n$ (equations 3 and 5). After 70 doses it will be about $10^{-8}$ of its original size, and continued therapy will eventually eliminate it altogether. The population of normal haemopoietic cells, on the other hand, should settle down to a steady-state level of about 0·75 of its normal size under continued therapy (equations 3 and 10). This regimen therefore has the required specificity. Now take an incorrectly chosen regimen, say, 33 mg./kg. given every 3 days. From equations (3), (5) and (10) it can be seen that this regimen eliminates the normal cells ($F = 0{\cdot}13 < e^{-kt} = 0{\cdot}22$), yet it allows the leukaemic cell population to grow, although at a slower rate than in an untreated individual (this population increases by a factor of 1·48 between each dose). It should be noted that, in this example, the overall dosage in a given time is the same as that in the regimen with the desired selectivity; only the size and frequency of the individual doses have been changed. Evidently, such simple changes in regimen may radically alter the selectivity of action of a drug, and may make all the difference between a treatment that is useful and one that is disastrous. The small probability of picking by chance a regimen with the required selectivity in this example is also clear from Fig. 5, and this is relevant, not only to therapy, but to the screening of new drugs.

Now consider the design of therapeutic regimen aimed at destroying a solid tumour growing according to equation (6), using an alkylating agent giving exponential dose-response curves with tumour cells and normal haemopoietic cells. It will be assumed that the dose-response curve for normal haemopoietic cells is that given by Bruce, Meeker and Valeriote (1966) for cyclophosphamide. This is a simple exponential curve without a shoulder, the value of $\alpha$ being 0·63. The value of $k$ will be taken as 0·5 as in the previous example. There are no adequate available data from which to derive the parameters of the dose-response curves for solid tumours; we shall here assume that the particular tumour cells in question are highly sensitive and give an exponential dose-response curve with a shoulder when treated with the agent, the value of $\alpha$ being 0·7 and of $\beta$, 5. Typical values for the Gompertz equation parameters $a$ and $b$ are 0·2 and 0·02 (Laird, 1964). The appropriate equation for elimination of the tumour is equation E in Table I, which gives

$$D > -\frac{1}{0{\cdot}7}\log_e\{1 - [1 - e^{-10(1-e^{-0{\cdot}02t})}]^{\frac{1}{5}}\} \qquad (12)$$

The appropriate equation for conservation of haemopoietic cells is equation G, which gives, for a required steady-state of not less than 0·5 of the normal level,

$$D < -\frac{1}{0\cdot 63}\log_e\left[\frac{2e^{-0\cdot 5t}}{1+e^{-0\cdot 5t}}\right] \qquad (13)$$

Values of $D$ against $t$ have been plotted for equations (12) and (13) in Fig. 6. Regimens with the required specificity are the high-dose, low-frequency regimens in the shaded area of the graph.

FIG. 6.—Design of selective regimens for a tumour obeying the Gompertz growth equation, using cyclophosphamide. The dose-response curve for normal haemopoietic cells (population B), has a simple exponential form (Bruce, Meeker and Valeriote, 1966), so equation G in Table I is used; $\alpha = 0\cdot 63$, $k = 0\cdot 5$ day$^{-1}$. It is assumed that the dose-response curve for tumour cells (population A) has a shoulder and that the relevant values (equation E, Table I) are $\alpha = 0\cdot 7$, $\beta = 5$, $a = 0\cdot 2$, $b = 0\cdot 02$. Regimens that will eliminate the tumour and conserve normal haemopoietic cells are in the shaded area.

In practice, relatively few cell populations can be expected to behave in the simple ways here described. Tissues such as the leucopoietic or erythropoietic fractions of the bone marrow have to be considered as multi-compartment systems and not homogeneous populations (Lajtha, Oliver and Gurney, 1962; Lajtha, Gilbert, Porteous and Alexanian, 1964; Lajtha, 1968). If, for example, the cells of a precursor compartment were more sensitive to the agent or recovered more slowly after depletion than the cells of the compartment being studied, the latter could be depleted far more by a prolonged dose regimen than would be suggested by consideration of its dose-response curve and recuperative properties after single doses of agent.

Considerable complexity would also be introduced by the existence of a non-growing fraction, such as occurs in many tumours (Mendelsohn, 1963). Inhomogeneity might also arise from the presence of a small fraction of proliferating cells relatively resistant to the agent, as is suggested, for example, by the tendency of the dose-response curves for methotrexate and thioguanine to flatten out at high doses (Berenbaum, 1969, Fig. 2). If this resistance is characteristic of a particular phase of the cell cycle it would probably not materially affect the considerations advanced here, provided the doses used were in the range where the dose-response curve is straight and the dosage interval was not in phase with the cell cycle (Merkle, Stuart and Gofman, 1965; Stuart and Merkle, 1965). If, on the other hand, high resistance were a genetic property of a particular fraction of the population, then the properties of the population would change during treatment, and dose-response curves based on experiments with single doses would become progressively less valid with increasing length of treatment.

There is evidently a need for a more sophisticated mathematical analysis than that outlined in this paper, and for more comprehensive experimental data on which to base it. Nevertheless, the simplified approach adopted here suggests that manipulations of the therapeutic schedule may influence not only the magnitude and speed of the effect of an agent on particular cell types but also its selectivity of action for these various types. The clinical relevance of this conclusion requires no emphasizing.

These considerations also underline the importance of the dose regimen in the clinical trial of antitumour and immunosuppressive agents. The examples shown in Fig. 5 and 6 suggest that some drugs rejected after poor performance in clinical trials might have appeared in a different light had different dose schedules been used. The practical problems involved in the satisfactory trial of a new agent are so large, however, that the thorough testing of a variety of dose schedules for each agent is hardly practicable with present resources. The alternative approach, which would supplement information obtained clinically, entails the intensive study of those cell characteristics responsible for selectivity of drug action, the determination of dose-response curves for cell types of clinical importance, and the measurement of other factors (generation times, growth and differentiating fractions, parameters of homoeostasis, etc.) that influence the overall effect of drugs on particular tissues. The difficulties of this approach are formidable, but the possibility of rationally designing therapeutic regimens of relatively high selectivity against neoplastic or immunologically active cells is sufficient inducement to attempt this task.

### SUMMARY

In a simplified model, the fate of growing or steady-state cell populations under a regimen of repeated doses of a cell-sterilizing agent may be predicted from equations containing two types of parameter—(1) those determined by the dose-response curve and the proliferative or recuperative characteristics of the cell populations and (2) those determined by the therapeutic regimen (i.e. size, frequency and number of doses). If the values of the former are known, those of the latter may be adjusted to ensure either destruction or survival of the cell population. Different cell types show different dose-response curves to the same agent and have different proliferative or recuperative rates. Regimens may therefore be chosen that selectively damage one cell population while allowing another in the same individual to survive. The model suggests that, in some circumstances, the fates of two cell populations in the same individual may be reversed by manipulating the therapeutic regimen.

I am indebted to the Leukaemia Research Fund, the Medical Research Council and the British Empire Cancer Campaign for Research for generous support.

### REFERENCES

BERENBAUM, M. C.—(1969) *Br. J. Cancer*, **23**, 426.

BRUCE, W. R. AND MEEKER, B. E.—(1967) *J. natn. Cancer Inst.*, **38**, 401.

BRUCE, W. R., MEEKER, B. E. AND VALERIOTE F. A.—(1966) *J. natn. Cancer Inst.* **37**, 233.

BUSH, W. R. AND BRUCE, W. R.—(1964) *Radiat. Res.*, **21**, 612.

KENNEDY, J. C., TILL, J. E., SIMINOVITCH, L. AND MCCULLOCH, E. A.—(1965) *J. Immunol.*, **94**, 715.

LAIRD, A. K.—(1964) *Br. J. Cancer*, **18**, 490.—(1965) *Br. J. Cancer*, **19**, 278.

LAJTHA, L. G.—(1968) *Radiat. Res.*, **33**, 659.

LAJTHA, L. G., OLIVER, R. AND GURNEY, C. W.—(1962) *Br. J. Haemat.*, **8**, 442.

LAJTHA, L. G., GILBERT, C. W., PORTEOUS, D. D. AND ALEXANIAN, R.—(1964) *Ann. N.Y. Acad. Sci.*, **113**, 742.

MENDELSOHN, M. L.—(1963) in ' Cell proliferation '. Edited by L. F. Lamerton and R. J. M. Fry. Oxford (Blackwell).

MERKLE, T. G., STUART, R. N. AND GOFMAN, J. W.—(1965) ' The calculation of treatment schedules for cancer chemotherapy ' UCRL—14505.

SACHER, G. A. AND TRUCCO, E.—(1966) *Radiat. Res.*, **29**, 236.

SKIPPER, H. E.—(1967) *Cancer Res.*, **27**, 2636.

SKIPPER, H. E., SCHABEL, F. M. JR. AND WILCOX, W. S.—(1964) *Cancer Chemother. Rep.*, **35**, 1.—(1965) *Cancer Chemother. Rep.*, **45**, 5.

STUART, R. N. AND MERKLE, T. C.—(1965) ' The calculation of treatment schedules for cancer chemotherapy—Part II ' UCRL—14505.